# Anti-Stokes fluorescence from endogenously formed protoporphyrin IX – Implications for clinical multiphoton diagnostics

**DOI:** 10.1002/jbio.201200119

**Published:** 2012-09-18

**Authors:** Despina Kantere, Stina Guldbrand, John Paoli, Mattias Goksör, Dag Hanstorp, Ann-Marie Wennberg, Maria Smedh, Marica B Ericson

**Affiliations:** 1 Department of Dermatology, University of GothenburgGothenburg, Sweden; 2 Department of Physics, University of GothenburgGothenburg, Sweden; 3 Centre for Cellular Imaging, the Sahlgrenska Academy, University of GothenburgGothenburg, Sweden

**Keywords:** two-photon fluorescence microscopy, aminolevulinic acid, methylaminolevulinate, protoporphyrin IX, skin cancer, anti-Stokes fluorescence

## Abstract

Multiphoton imaging based on two-photon excitation is making its way into the clinics, particularly for skin cancer diagnostics. It has been suggested that endogenously formed protoporphyrin IX (PpIX) induced by aminolevulinic acid or methylaminolevulinate can be applied to improve tumor contrast, in connection to imaging of tissue autofluorescence. However, previous reports are limited to cell studies and data from tissue are scarce. No report shows conclusive evidence that endogenously formed PpIX increases tumor contrast when performing multiphoton imaging in the clinical situation. We here demonstrate by spectral analysis that two-photon excitation of endogenously formed PpIX does not provide additional contrast in superficial basal cell carcinomas. In fact, the PpIX signal is overshadowed by the autofluorescent background. The results show that PpIX should be excited at a wavelength giving rise to one-photon anti-Stokes fluorescence, to overcome the autofluorescent background. Thus, this study reports on a plausible method, which can be implemented for clinical investigations on endogenously formed PpIX using multiphoton microscopy.

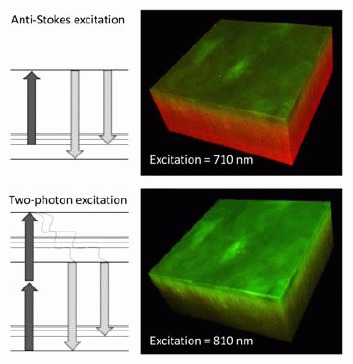

Three-dimensional multiphoton microscopy images obtained from a superficial basal cell carcinoma illustrating higher porphyrin contrast when anti-stokes excitation (710 nm) is used compared to two-photon excitation (810 nm).

## 1. Introduction

Non-linear optical microscopy is becoming an important tool in the biosciences [1, 2]. In particular, a technique based on two-photon excitation of tissue autofluorescence using near-infrared radiation (NIR) has shown to be a powerful tool for obtaining non-invasive tissue biopsies of skin tumors. The technology is making its way into the clinics launched as multiphoton tomography [3, 4]. But despite promising results for tumor diagnostics based on tissue autofluorescence [5, 6], there is a continued quest for suitable contrast agents, providing elevated tumor contrast. As aminolevulinic acid (ALA) or its methylester methylaminolevulinate (MAL) have been applied to provide tumor contrast for macroscopic fluorescence imaging of human skin tumors [7–9], it comes as a natural step that these drugs could be useful in providing tumor contrast also for multiphoton imaging. This has indeed been sug-gested by others [10, 11]. Preliminary data exist, but reports are mainly restricted to cell cultures [12–15]. Data from skin tumors are limited and lack thorough spectral investigations [11, 15].

When ALA or MAL is applied to living tissue, they metabolize to form protoporphyrin IX (PpIX) through the heme biosynthesis pathway [16]. PpIX is a fluorescent porphyrin, which is well characterized when it comes to its one-photon excitation properties [17, 18], exhibiting a strong absorption peak in the blue region, i.e. 405 nm, known as the Soret-band, due to the transition from the ground singlet state to the second electronic singlet state. However, detailed studies of two-photon excitation of PpIX are scarce. There are reports on multiphoton excitation of PpIX using NIR light in solution [19], gastrointestinal cancer tissue, cell cultures [12–14] and mouse tissue [20, 21]. A few studies report on multi-photon excitation of endogenously formed PpIX in skin tissue [11, 15], but detailed spectral investigations are lacking.

We report on an investigative study with the aim to set up a method for multiphoton imaging of non-melanoma skin cancer, e.g. basal cell carcinoma, using MAL-induced PpIX fluorescence. We present a spectral investigation of two-photon excitation and emission of PpIX in solution, and from excised skin tumors from patients with basal cell carcinoma exposed to MAL. Our results demonstrate that two-photon excitation of PpIX in skin does not improve tumor contrast. Instead, we present a method, based on NIR one-photon anti-Stokes excitation of PpIX, which makes it possible to visualize endogenously formed PpIX in skin tumors.

## 2. Experimental

### 2.1 Chemicals

Protoporphyrin IX (MW = 562.658 g/mol, purity of ≥95%, Sigma-Aldrich Sweden AB) was dissolved in dimethyl sulfoxide (DMSO, purity of ≥99.5%, Sigma-Aldrich Sweden AB) for the measurement of single- and two-photon excitation spectra. For the clinical study, MAL- cream (METVIX®, Galderma, 160 mg/g methyl aminolevulinate as hydrochloride), and placebo cream (Unguentum M, Hermal, Rein-bek, Germany) were used.

### 2.2 Skin samples

The patients included in this study account for a subset from a major clinical trial at the Dept. of Dermatology, Sahlgrenska University Hospital, Gothenburg, approved by the local ethics committee (Dnr: 229-09). Initially nine patients with non-melanoma skin cancer were included and treated with ALA or MAL according to protocol. The lesions were imaged using 780 nm excitation with equipment as described earlier [5]; however, no difference between autofluorescence and the PpIX channel were observed, and the study was aborted. Instead, an investigative study was initiated including three additional patients with histopathologically verified superficial basal cell carcinomas (for demography, see Supplementary methods). In this work, data from these three patients are presented. The patients gave written, informed consent prior to inclusion. MAL cream was applied to the lesions of two patients for 3 hours according to clinical routine. Placebo cream was applied to one lesion in the same manner. The application areas were covered with an occlusive and light-protective dressing.

Biopsies from both the tumour bulk and perilesional normal skin were obtained, using a 6 mm diameter dermal biopsy punch (Miltex Inc., York, PA). The biopsies were trimmed eliminating a part of sub-cutaneous tissue with surgical scissors. Half of the tissue biopsies obtained from one of the MAL treated patients were cryosectioned in 10 mm thin tissue sections and mounted on microscope slides, while the other half was stored in a freezer (−70 °C). The full thickness skin biopsies were placed in an imaging chamber gasket with the skin surface against the cover glass and mounted on a microscope slide. The slides with the prepared specimens were wrapped in aluminium foil to protect them from light and stored in a freezer (−18 °C) for approximately 1 hour until imaging began.

### 2.3 Multiphoton set-up

The multiphoton set-up was a LSM 710 NLO microscope system (Carl Zeiss MicroImaging GmbH, Germany), connected to a Mai Tai DeepSee tunable NIR Ti:Sapphire fs-laser (Spectra-Physics, Newport Corporation, USA). A Plan-Apochromat 20×/1.0 water immersion objective, corrected for a 0.17 mm cover glass, having a working distance of 1.9 mm, was used in all experiments.

For the spectral measurements in solution, a droplet of the solution was placed between two cover-slips. The emission spectra were obtained by exciting the sample either using a 405 nm diode laser or the NIR fs-laser operating at 710 nm or 810 nm, and the emission was recorded between 400-700 nm with steps of 10 nm using the internal LSM 710 spectral detector. The excitation spectra were obtained by sequentially changing the excitation wavelength in the range of 700–950 nm with steps of 10 nm and the corresponding emission signal was detected in the range of 610–680 nm. The excitation spectra were acquired both increasing and decreasing wavelengths, in order to avoid hysteresis.

The same microscope was used for both multiphoton and confocal fluorescence imaging, using either the NIR fs-laser (710 nm or 780 nm) or the diode laser (405 nm). A pinhole of 1 airy unit was used for confocal imaging, whereas NIR fs-laser imaging was performed with an open pinhole (if not otherwise stated). For regular channel mode images, the skin autofluorescence was recorded in the emission range of 410–580 nm, and the PpIX emission in the range of 610–690 nm using the descanned pathway.

## 3. Results

### 3.1 Near-infrared fs-laser excitation results in one-photon anti-Stokes emission of PpIX

**Figure 1:**
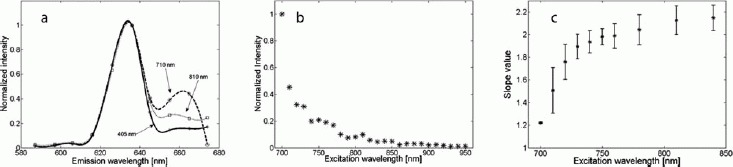
**(a)** Emission spectra of protoporphyrin IX in dimethylformamide solution (2.6 mM) normalized at the Q(0,0) emission-peak acquired using different excitation wavelengths. **(b)** Near infrared excitation spectra of the same solution (emission detected in the range of 600–690 nm). **(c)** Investigation of power-dependency of the PpIX fluorescence as a function of wavelength (See also Supplementary Figure S1). Data presented as mean values obtained from 10 measurements at each wavelength. Errorbars correspond to the standard deviation.

One-photon absorption and excitation of porphyrins using light in the visible wavelength range are well understood [17, 22], but data from two-photon excitation using highly focused NIR fs-laser light of porphyrins are surprisingly scarce. Figure 1a shows the emission spectrum of PpIX in solution acquired at three different excitation wavelengths. As shown by the figure, the characteristic Q(0,0) emission peak centered at 630 nm is evident for all excitations, both 405 nm and NIR two-photon excitation at 710 nm and 810 nm. It should be noted that a stronger contribution to the emission around 670 nm is obtained for the 710 nm excitation. This emission most likely corresponds to presence of photoproduct (i.e. photo-PpIX), which is known to have emission in this region [19, 23]. By increasing the energy of the excitation light, the emission from the photo-PpIX will increase because of a better matching of its Soret-transition.

**Figure 2:**
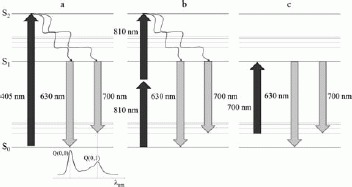
Jablonski diagram illustrating three possible processes for protoporphyrin IX excitation, **(a)** one-photon excitation (405 nm), **(b)** two-photon excitation (810 nm), and **(c)** anti-Stokes excitation (700 nm) from the first vibronic level of *S*_0_. Bold arrows represent absorption; grey arrows, emission; and curved arrows, radiationless transitions. The emission peaks at 630 nm and 700 nm correspond to the Q(0,0) and Q(0,1) transitions respectively, as illustrated by the schematic emission spectrum insert.

Figure 1b demonstrates the excitation spectrum of a solution of PpIX when excited with fs-pulsed NIR light. As shown by the figure, the fluorescence intensity drastically increases when the excitation wavelength decreases and approaches 700 nm. This absorption behavior has earlier been observed for other porphyrins by Drobizhev et al. [24], who explained this increase by a two-photon resonance enhancement due to the nearby Q(0,0) transition. However, for pure two-photon excitation, the emission intensity should be proportional to the square of the power of the excitation light. When investigating the dependency of the PpIX fluorescence signal with respect to the laser power (Figure 1c and Supplementary Figure S1), it was found that for shorter wavelengths approaching 700 nm there is a gradual transition of the non-linearity of the excitation process into a linear process. Thus, the increased excitation probability cannot be fully explained by a nonlinear resonance effect. Instead, we show that this phenomenon is most likely caused by an increased probability of anti-Stokes one-photon absorption, illustrated by Figure 2.

It is well known that porphyrins exhibit a Q(0,1) transition in their emission spectrum, but reports on one-photon absorption corresponding to this transition are lacking, because of the low population of the first vibronic energy level in the ground state. However, when using highly focused fs-laser NIR light for excitation of porphyrins, the unlikely two-photon excitation process, i.e. having a cross-section (σ_2PE_) of the order of 10^−50^ cm^4^ s, photon, competes with a more probable one-photon transition, i.e. cross-section (σ_1PE_) of the order of 10^−15^ cm^2^ photon [25]. The probabilities of the one-photon (*P*_1PE_) and the two-photon (*P*_2PE_) excitation processes can be described as


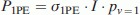



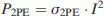


where *I* is the photon flux in the focus, and *p*_*v*=1_ is the thermal population, i.e. the population-ratio of the first vibronic level. Given the experimental conditions in our set-up (Supplementary material), the thermal population, i.e. the population-ratio of the first vibronic level, needs to be larger than 10^−4^ for the one-photon process to take over. An estimation using Boltzmann's equation,


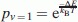


results in a thermal population of 5 × 10^−4^ of the first vibronic level of PpIX at room temperature, explaining why one-photon anti-Stokes fluorescence can be observed. In addition, there will be a buildup of PpIX in its first vibronically excited, but electronical ground state as a consequence of the excitation/deactivation process via the Q(0,1) transition. Emission via the Q(0,1) transition will leave the molecules in the first vibronic level during the fs-pulse before vibrational relaxation, as earlier demonstrated for iron porphyrins and heme using Raman spectroscopy [26, 27]. Due to the high momentary density of photons in the fs-laser pulses, it is likely that a non-equilibrium of excited vibrational energy distribution will occur, contributing to an increased probability for anti-stokes excitation. Furthermore, the fs-laser pulses (approx. 60 fs) have a spectral bandwidth of around 14 nm, leading to a gradual transition to one-photon anti-Stokes fluorescence as the wavelength of the excitation light is decreased. Taken together, our spectral investigation demonstrates that when exciting PpIX using high-intensity, fs-pulsed NIR excitation, the contribution of one-photon anti-Stokes fluorescence gradually takes over from non-linear excitation, when decreasing the wavelength of the excitation light close to 700 nm.

### 3.2 Lack of specific non-linear excitation of endogenous PpIX

**Figure 3:**
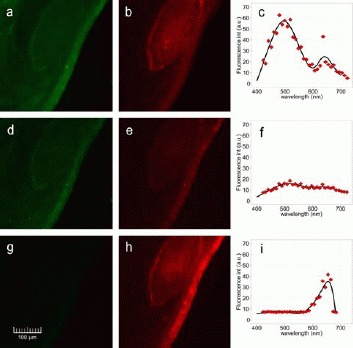
Laser scanning confocal images and multiphoton excitation images of cryosections (10 μm thickness) of a superficial basal cell carcinoma, exposed to MAL 3 hours before excision. The left panel shows the green autofluores-cence channel; the center panel, the red PpIX channel; and the right panel, the emission spectrum obtained from the same section. The first row demonstrates data obtained using one-photon excitation (405 nm), the second row shows two-photon excitation (810 nm) and the third row shows anti-Stokes excitation (710 nm). Scalebar equals 100 μm (same for all images).

Our first attempts to visualize endogenously formed PpIX using two-photon excitation and similar settings as earlier reported, i.e. using fluorescence excitation in the range of 750–800 nm [11, 15], did not reveal any specific fluorescence signal from PpIX. Therefore, a detailed study of the excitation and emission of endogenously formed PpIX was undertaken. Figure 3 presents fluorescence images obtained from a cryosection of an excised superficial basal cell carcinoma previously exposed to MAL for 3 hours in vivo. Presence of PpIX in the lesion was confirmed by fluorescence imaging before excision (Supplementary Figure S2). Included in Figure 3 is also the fluorescence emission spectra obtained from the same sample. The characteristic porphyrin peak in the emission spectrum (Figure 3c) using 405 nm one-photon excitation confirms that PpIX is present. The autofluorescence emission (Figure 3a) and PpIX emission (Figure 3b) can easily be separated by choosing the correct emission settings using 405 nm excitation. Interestingly, no evident porphyrin peak can be discerned from the spectrum when NIR excitation, i.e. 810 nm, is applied (Figure 3f). Instead the PpIX emission seems engulfed in a broadening of the autofluorescence spectrum. This is also evident from the fluorescence images, where overlapping features are observed in the autofluorescence (Figure 3d) and the PpIX channel (Figure 3e). Thus, the distribution of endogenously formed PpIX cannot be easily discerned using multiphoton NIR excitation in skin tumors. On the other hand, when the excitation wavelength is lowered to 710 nm, i.e. a wavelength where the likelihood for one-photon anti-Stokes excitation is increased, the PpIX emission is significantly increased compared to the autofluorescence signal (Figure 3i). Due to a slight shift in emission peak, the contribution of photo-PpIX seems to be elevated. Taken together, at this excitation wavelength, the PpIX signal can be discerned from the autofluorescence (Figure 3h vs. Figure 3g).

### 3.3 3D-visualization of PpIX in skin based on anti-Stokes fluorescence

**Figure 4:**
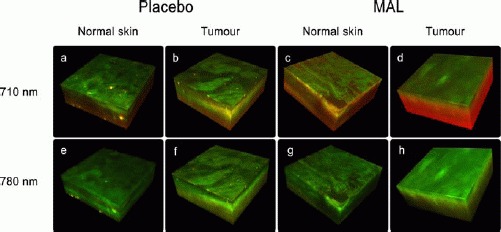
Three-dimensional reconstruction of multiphoton microscopy z-stacks obtained from two different superficial basal cell carcinomas (**b, f**, and **d, h**) and the corresponding surrounding normal skin (**a, e** and **c, g**). In the upper row, anti-stokes 710 nm excitation was used, and in the bottom row, 780 nm. The lesions had either been exposed to placebo (**a, e**, and **b, f**) or to MAL (**c, g** and **d, h**). Field of view for each image: 213 × 213 × 90 μm.

Intact superficial basal cell carcinomas and perile-sional healthy skin exposed to MAL *in vivo* for 3 hours were excised and examined *ex vivo* performing three-dimensional multiphoton microscopy. Imaging was performed on skin tumors and perilesional healthy skin with placebo for comparison. No specific PpIX fluorescence (red channel) was observed when 780 nm excitation was applied, neither after the application of MAL, nor placebo, as demonstrated by Figure 4 (and Supplementary Figures S3–S6). Predominantly the skin autofluorescence was registered using this excitation wavelength. On the other hand, when 710 nm excitation was used, distinct differences between the autofluorescence and the red channel were observed, particularly after MAL application. Evident from the figure is also the higher accumulation of PpIX in the tumor compared to the surrounding tissue, a feature that could not be observed using 780 nm excitation. As 710 nm excitation results in a one-photon process, the signal in the red channel has lower resolution compared to the autofluorescence. Still, the anti-Stokes fluorescence is superior compared to conventional one-photon excitation, as only very limited signal from the outmost layers of the epidermis could be obtained using 405 nm excitation (data not shown). Also, a spectral investigation confirmed that PpIX emission from intact skin could only be obtained using 710 nm excitation (Supplementary Figure S7). Thus, in order to visualize PpIX induced by MAL in skin tumors, one-photon anti-Stokes excitation is preferred.

## 4. Discussion

Multiphoton imaging is becoming a tool for non-invasive clinical diagnostics for a variety of diseases, with a special focus on skin cancer [4–6]. It is well known that skin tumors accumulate PpIX after application of ALA or MAL [28]. Endogenously formed PpIX has been suggested to provide enhanced tumor contrast in combination with multiphoton imaging [10, 11, 15], but earlier data are scarce. We found that pure multiphoton excitation of endogenously formed PpIX does not provide contrast in skin tumours. Instead PpIX should be excited at a wavelength close to 700 nm giving rise to anti-Stokes fluorescence, which is possible due to the thermal population of the first vibronic state of PpIX.

The two-photon cross section has earlier been re-ported to be low for PpIX [19], explained by unfavorable multiphoton excitation of the Soret band. Exact two-photon excitation cross sections of tissue autofluorescence are difficult to obtain, as the concentration of the fluorophores are unknown. Despite the fact that fluorescence from NADH and flavins seem to increase with decreasing wavelength [29, 30], our results imply that shorter wavelengths are preferred in order for PpIX to overcome the autofluorescent background. As one-photon anti-Stokes excitation is a linear process, the confinement of excitation is lost and the out-of-focus signal increases. This was also observed as a deterioration of the resolution in our study. In principle, this could be corrected by introducing a pinhole. In our set-up, we could not improve the resolution by introducing a pinhole as the PpIX signal was diminished. Thus, there is a trade-off between the signal and resolution of the PpIX fluorescence.

There are several reports on one-photon excitation for visualizing skin tumors based on 405 nm excitation of endogenously formed PpIX [7, 31, 32], restricted to macroscopic surface fluorescence. In order to improve resolution and imaging depth, multi-photon imaging has been suggested. We here demonstrate that pure two-photon excitation of PpIX in skin lesions is not possible, and instead anti-Stokes excitation using 710 nm excitation should be used. Compared to 405 nm excitation, the results demonstrate that the 710 nm anti-Stokes excitation is superior when it comes to imaging depth with cellular resolution in a confocal microscope, but a NIR fs-pulsed laser is required. Even though the resolution of anti-Stokes excited PpIX is not the same as in pure two-photon excitation, the distribution of tissue PpIX can be discerned. By combining with two-photon excitation of autofluorescence, the cellular morphology can be visualized.

Endogenously formed PpIX is perhaps most widely applied for therapeutic purposes, i.e. photodynamic therapy (PDT), rather than for diagnostics. It has also been suggested that endogenous PpIX in combination with multiphoton excitation could be used for confined PDT, based on the findings that the photochemistry and subsequent photobleaching using two-photon excitation of PpIX follows the same pathways as for one-photon excitation [19]. Earlier studies of PpIX emission using multiphoton excitation have been limited to solutions or cell lines [12–14] with less endogenous fluorescence compared to tissue. We here demonstrate that the emission based on two-photon excitation of endogenously formed PpIX is low. It is therefore likely that also the multiphoton photodynamic efficiency in tissue would be poor. Photobleaching is strongly related to the photodynamic effect and presence of oxygen [23, 33], and has been proposed as a tool to monitor multiphoton PDT [13]. Thus, our results also demonstrate the importance of investigating the photodynamic effect comparing multiphoton excitation and anti-Stokes excitation within the tissue.

To conclude, we show that skin cancers undergoing multiphoton imaging based on endogenously formed PpIX should be investigated on the verge of one-photon anti-Stokes fluorescence, to provide contrast between PpIX and the tissue autofluorescence. These results are important to report since other authors have reported ongoing clinical trials on multiphoton tomography in conjunct to ALA-induced PpIX in skin tumors, and should avoid extensive patient inclusion if correct settings are not used. Based on our findings, this new knowledge should be considered when implementing clinical investigations of endogenously formed PpIX using non-linear optical microscopy, providing the means for continued work in the field.

## Author biographies

Please see Supporting Information online.

## Supplementary material

As a service to our authors and readers, this journal provides supporting information supplied by the authors. Such materials are peer reviewed and may be re-organized for online delivery, but are not copy-edited or typeset. Technical support issues arising from supporting information (other than missing files) should be addressed to the authors.
