# Supplementary material for: Anti-Stokes fluorescence from endogenously formed protoporphyrin IX – Implications for clinical multiphoton diagnostics
Source: J Biophotonics. 2012 Sep 18;6(5):409–15. doi: 10.1002/jbio.201200119 (PMC3732385; doi:10.1002/jbio.201200119)
Supplement: Supplementary file 1 [file jbio0006-0409-SD1.pdf]

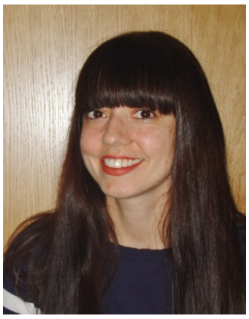

Despina Kantere was born in Greece. She received her MD degree in 2003 from the University of Athens. She is presently working as specialist in Dermatology and Venereology at the Sahlgrenska University Hospital, Gothenburg, Sweden. Her research field is skin tumors and two-photon fluorescence microscopy.

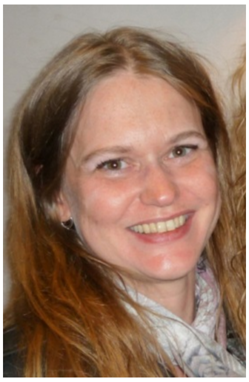

Stina Guldbrand is a PhD student in the Group of Biomedical Photonics at the Department of Physics, University of Gothenburg, Sweden. Her field of research involves studying the penetration and diffusion of fluorophores and nanoparticles in human skin by using two-photon fluorescence microscopy and fluorescence correlation spectroscopy.

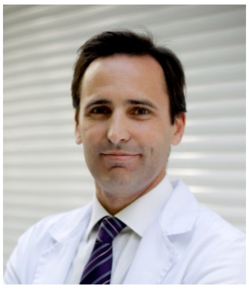

John Paoli received his MD degree from Universidad Miguel Hernández in Alicante, Spain in 2001. He specialized in Dermatology at Sahlgrenska University Hospital in Gothenburg, Sweden and headed the Skin Cancer and Surgery Center from 2007. Paoli obtained his PhD in 2009. He is currently Vice President of the Swedish Association of Dermatology and Venereology, President of the Swedish Association for Dermatologic Surgery and Oncology and a Board Member of the International Dermoscopy Society.

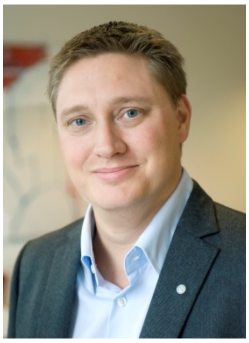

Mattias Goksör is the head of the Department of Physics at the University of Gothenburg. He is also manager for the research facility “Centre for Biophysical Imaging” and group leader for the Biophotonics research group. Goksör is the chairman of the Biological and Medical Physics section within the Swedish Physical Society and has founded one spin-off company dedicated for single cell analysis in cancer diagnostics.

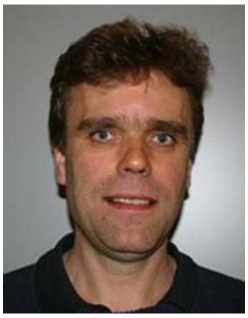

Dag Hanstorp studied mathematics, physics and didactics at the University of Gothenburg, Sweden where he received his PhD He is currently a professor in Experimental Physics at the University of Gothenburg. His research is conducted in the fields of atomic and molecular physics, applied laser spectroscopy and mass spectrometry.

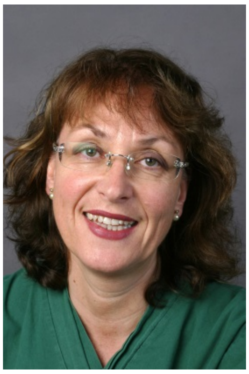

Ann-Marie Wennberg is Professor and MD in Dermatology and Venereology at the Sahlgrenska Academy and Sahlgrenska University Hospital in Gothenburg, Sweden. Wennberg holds a position as vice CEO at Sahlgrenska University Hospital and was the Head of the Department of Dermatology at Sahlgrenska University Hospital during 2007-2009. She is on the Advisory Board for the Swedish Cancer Society and a member of the Swedish Melanoma Study Group. She has been the President of the Nordic PDT group since 2004.

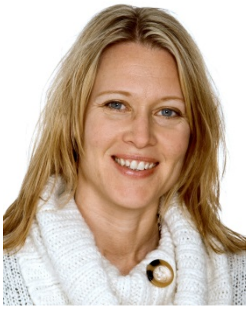

Maria Smedh received her degree in physics in 1996 from the University of Lund, where she also did her graduate work on surface science using different spectroscopic and microscopic methods. She obtained her PhD in physics in 2001 and continued working within the field of surface science 2001–2003 as a Post Doc at the University of Washington in Seattle, USA. In 2003 she received her current position at the advanced light microscopy core facility Centre for Cellular Imaging, the Sahlgrenska Academy, University of Gothenburg.

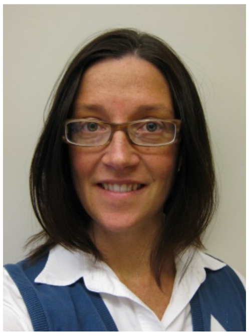

Marica B. Ericson is appointed Associate Professor at Dept. of Physics, University of Gothenburg, Sweden. Ericson is the principal investigator of the Biomedical Photonics group, and her research focusses on using advanced optical microscopy techniques for studies of the skin. Ericson's competence ranges from fundamental basic physical science and optical spectroscopy, to clinical applications. She received her PhD in Physical Chemistry from Chalmers University of Technology, Sweden, in 2004. She spent two years as PostDoc at the Department of Dermatology, Sahlgrenska University Hospital, Sweden, and has been visiting researcher at the University of Texas, Austin (TX), USA, 2010-2011, exploring nanoparticles and non-linear optical microscopy for biomedical applications.
