# Supplementary material for: Anti-Stokes fluorescence from endogenously formed protoporphyrin IX – Implications for clinical multiphoton diagnostics
Source: J Biophotonics. 2012 Sep 18;6(5):409–15. doi: 10.1002/jbio.201200119 (PMC3732385; doi:10.1002/jbio.201200119)
Supplement: Supplementary file 2 [file jbio0006-0409-SD2.pdf]

Supplementary material

Anti-Stokes fluorescence from endogenously formed protoporphyrin IX – Implications for clinical multiphoton diagnostics

Despina Kantere, Stina Guldbrand, John Paoli, Mattias Goksör, Dag Hanstorp, Ann-Marie Wennberg, Maria Smedh, and Marica B. Ericson

- **Supplementary methods**
- **Supplementary data**
- **Supplementary probability estimation**

Supplementary methods

Table S1. Demography of included patients

Patient code	Gender	Age	Histologically verified tumor type	Localization	Tumor diameter according to inclusion criterion	Applied drug
110	Male	72	Superficial basal cell carcinoma	Neck	> 10 mm	MAL
111	Female	67	Superficial basal cell carcinoma	Lower leg	> 10 mm	Placebo
112	Female	74	Superficial basal cell carcinoma	Lower leg	> 10 mm	MAL

Supplementary data

Supplementary Figure S1

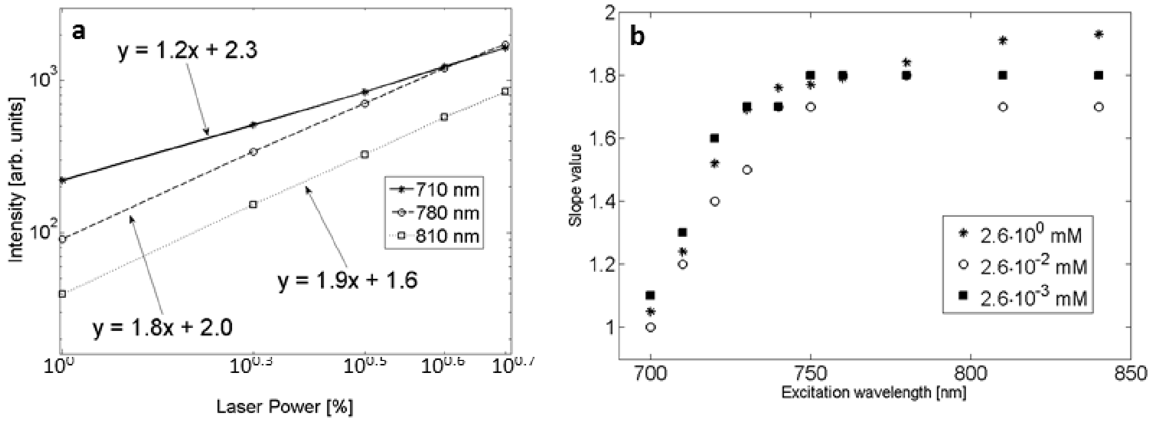

Figure S1. (a) Emission intensity of protoporphyrin IX in dimethylformamide solution (c = 2.6 mM) as a function of the laser power using different excitation wavelengths. Included are also the acquired slope-values for the different excitation wavelengths. (b) Power dependency, i.e. slope values, obtained for different excitation wavelengths at three different concentrations.

Supplementary Figure S2

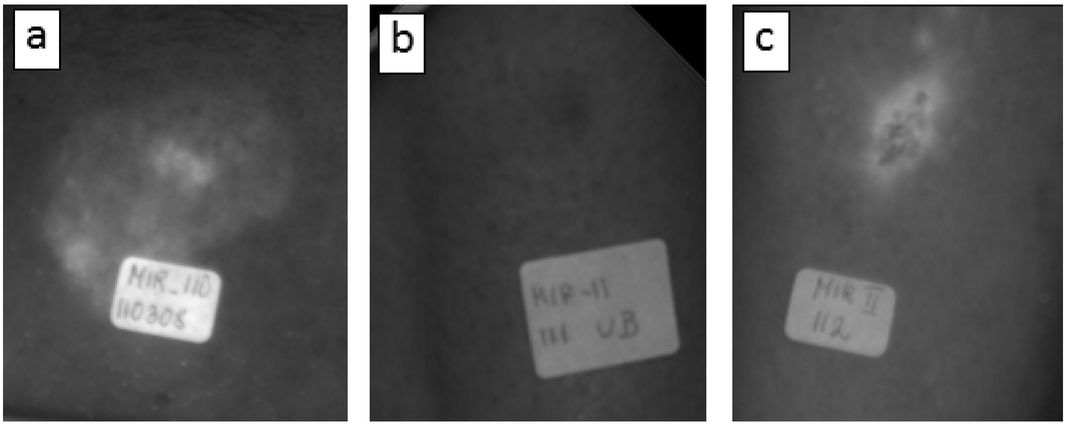

Figure S2. Macroscopic PpIX fluorescence images of lesions exposed to MAL (a,c) and placebo (b). The label width corresponds to 2 cm.

Supplementary Figure S3

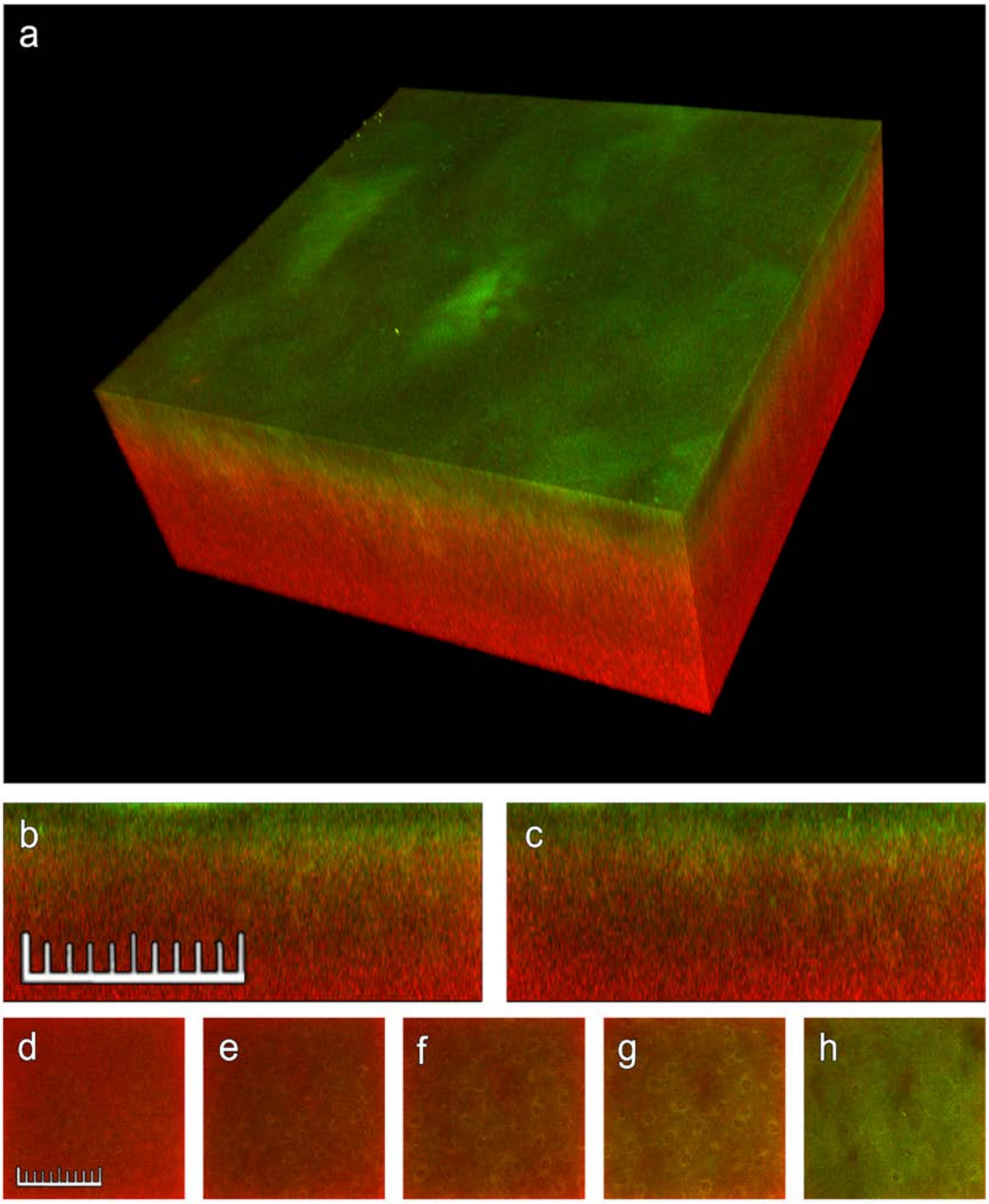

Figure S3. Multiphoton images presented as z-stack of a superficial basal cell carcinoma exposed to MAL. Imaging performed using 710 nm excitation. The red channel corresponds to protoporphyrin IX fluorescence, and the green channel corresponds to autofluorescence. (a) Three-dimensional reconstruction with a field of view: 213 x 213 x 90 µm, (b) and (c) are x,z and y,z optical vertical crossections respectively; (d-h) are optical horizontal crossections obtained at (d) z=76 µm, (e) z=62 µm, (f) z=48 µm, (g) z=34 µm, and (h) z=20 µm. Scalebar represents 100 µm.

Supplementary Figure S4

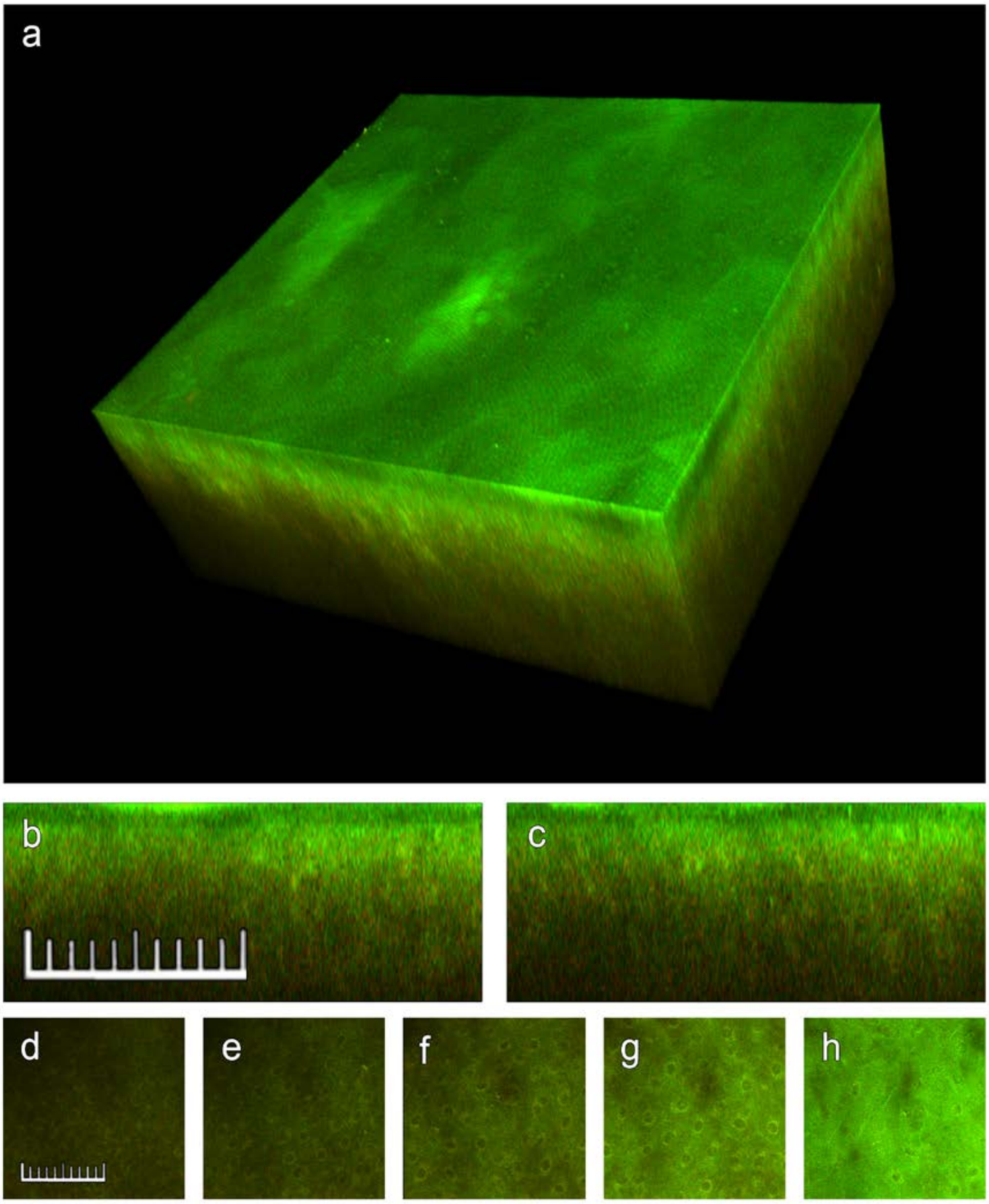

Figure S4. Multiphoton images presented as z-stack of the same lesion as in Figure S2. Imaging performed using 780 nm excitation. The red channel corresponds to protoporphyrin IX fluorescence, and the green channel corresponds to autofluorescence. (a) Three-dimensional reconstruction with a field of view: 213 x 213 x 90 μm, (b) and (c) are x,z and y,z optical vertical crossections respectively. (d-h) are optical horizontal crossections obtained at (d) z=76 μm, (e) z=62 μm, (f) z=48 μm, (g) z=34 μm, and (h) z=20 μm. Scalebar represents 100 μm.

Supplementary Figure S5

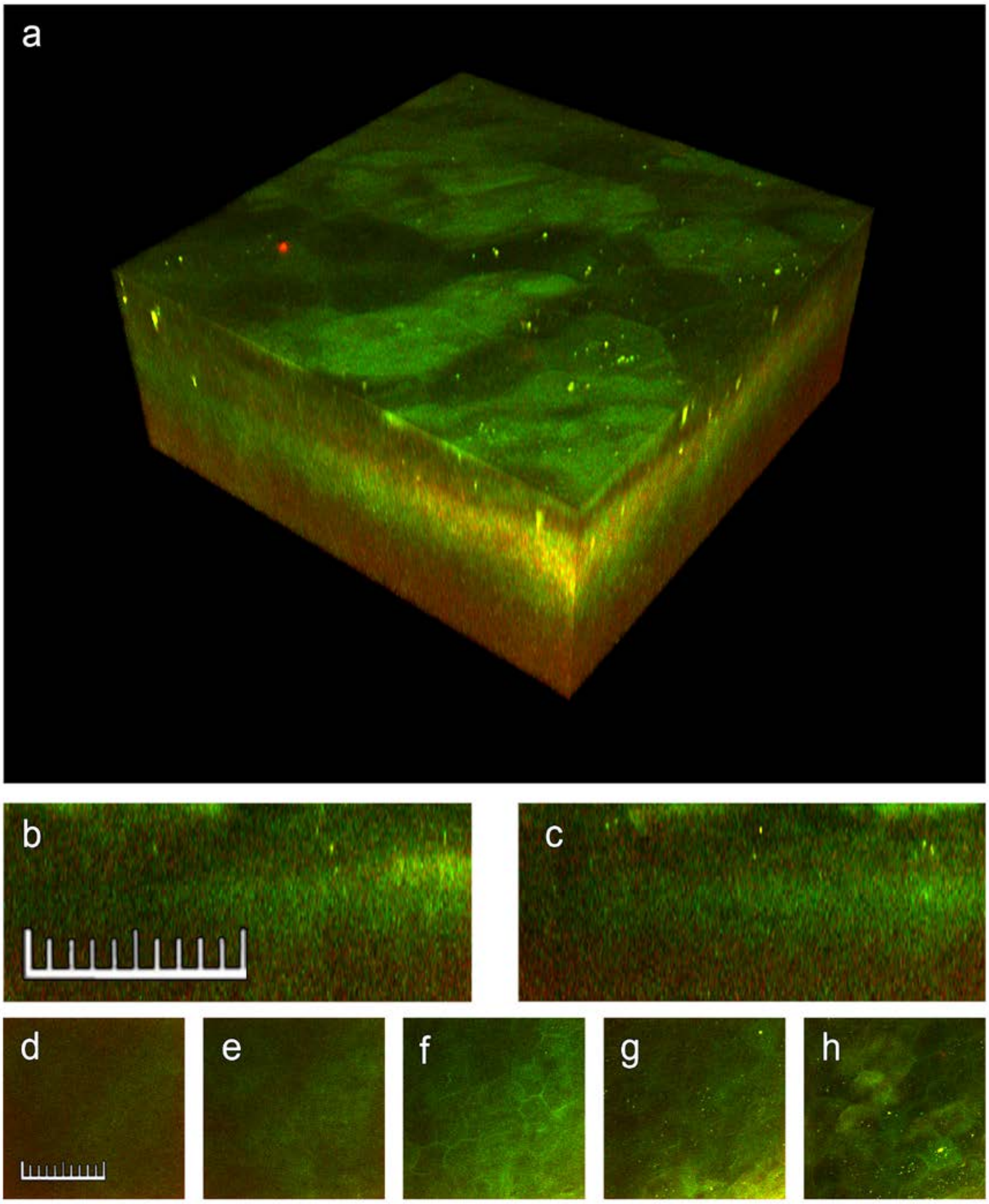

Figure S5. Multiphoton images presented as z-stack of a superficial basal cell carcinoma exposed to placebo. Imaging performed using 710 nm excitation. The red channel corresponds to protoporphyrin IX fluorescence, and the green channel corresponds to autofluorescence. (a) Three-dimensional reconstruction with a field of view: 213 x 213 x 92 µm, (b) and (c) are x,z and y,z optical vertical crossections respectively. (d-h) are optical horizontal crossections obtained at (d) z=86 µm, (e) z=68 µm, (f) z=50 µm, (g) z=32 µm, and (h) z=14 µm. Scalebar represents 100 µm.

Supplementary Figure S6

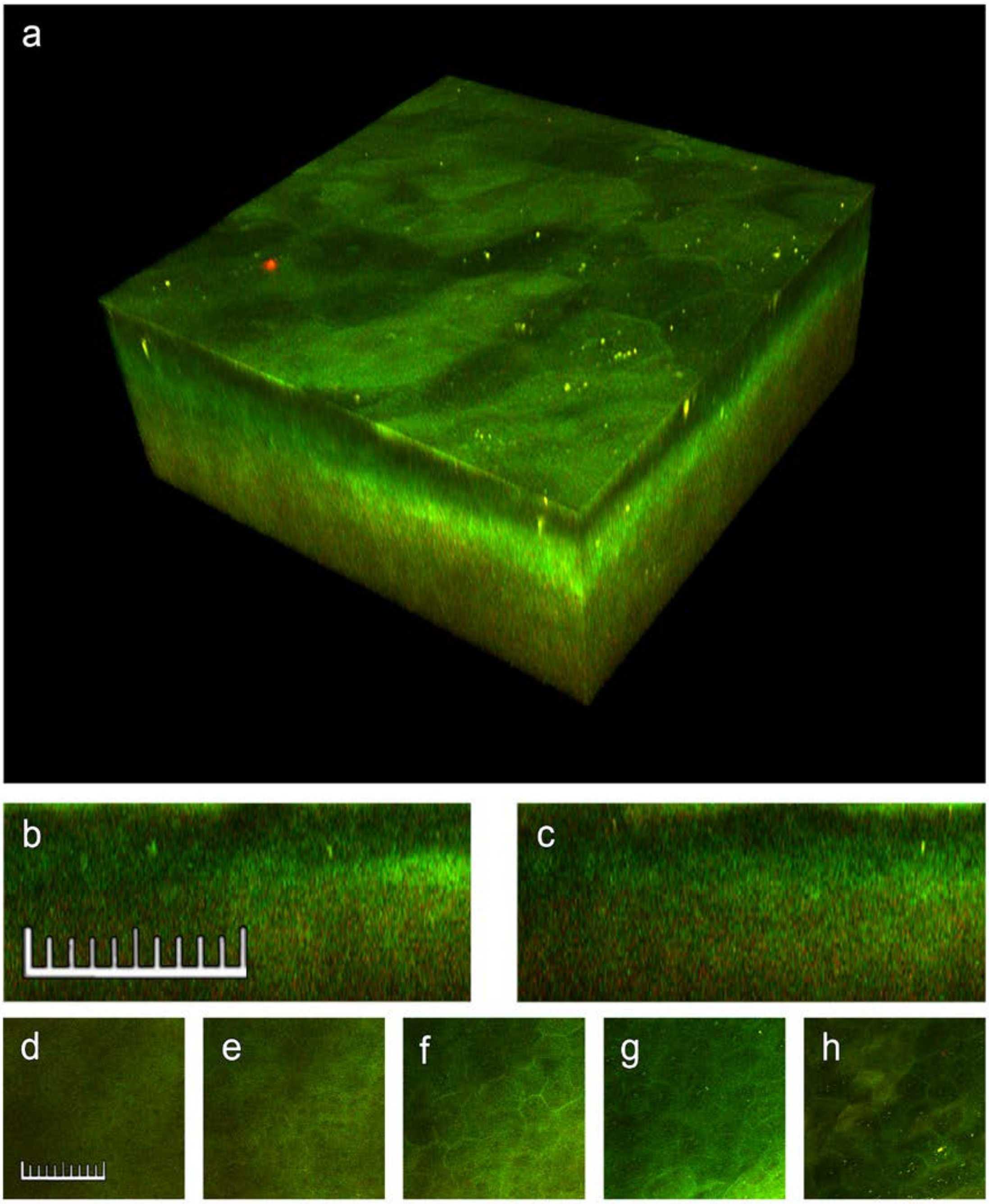

Figure S6. Multiphoton images presented as z-stack of same lesion as in Figure S4. Imaging performed using 780 nm excitation. The red channel corresponds to protoporphyrin IX fluorescence, and the green channel corresponds to autofluorescence. (a) Three-dimensional reconstruction with a field of view: 213 x 213 x 92 µm, (b) and (c) are x,z and y,z optical vertical crossections respectively. (d-h) are optical horizontal crossections obtained at (d) z=86 µm, (e) z=68 µm, (f) z=50 µm, (g) z=32 µm, and (h) z=14 µm. Scalebar represents 100 µm.

Supplementary Figure S7

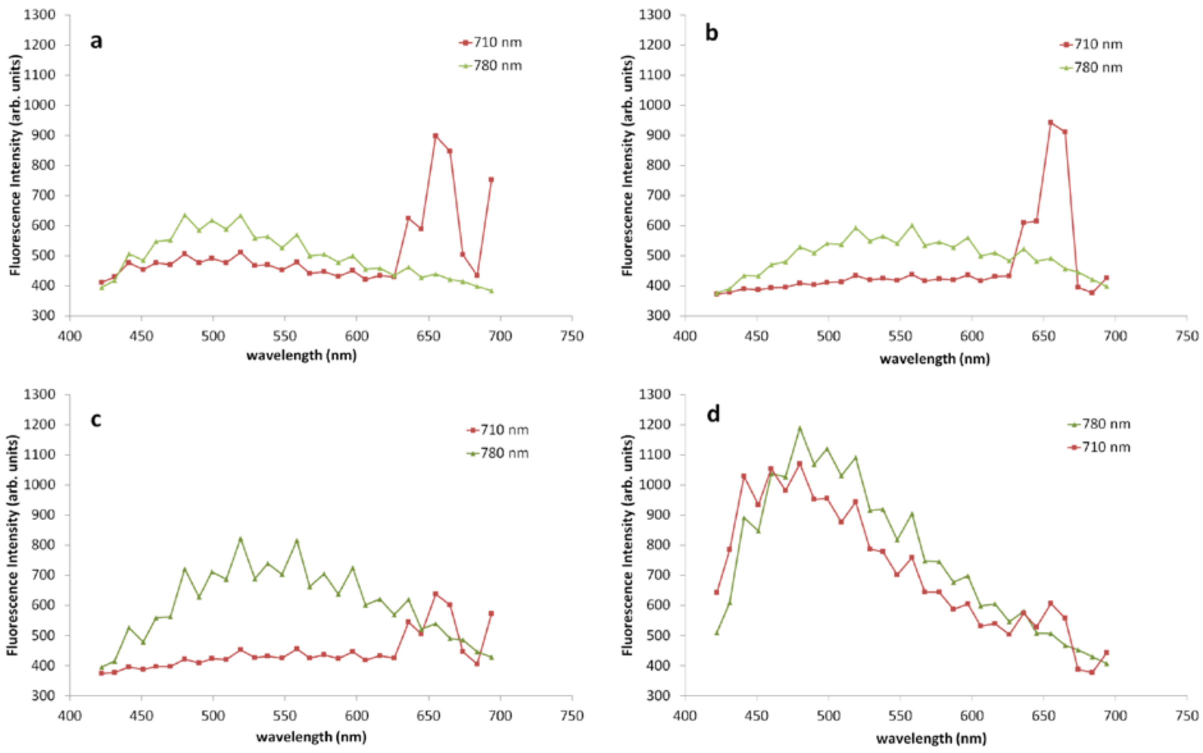

Figure S7. Emission spectrum obtained using 710 nm and 780 nm NIR excitation obtained from (a) perilesional skin exposed to MAL, (b) in basal cell carcinoma exposed to MAL, (c) perilesional skin exposed to placebo, and (d) basal cell carcinoma exposed to placebo. All spectra acquired using similar settings.

Supplementary probability estimation

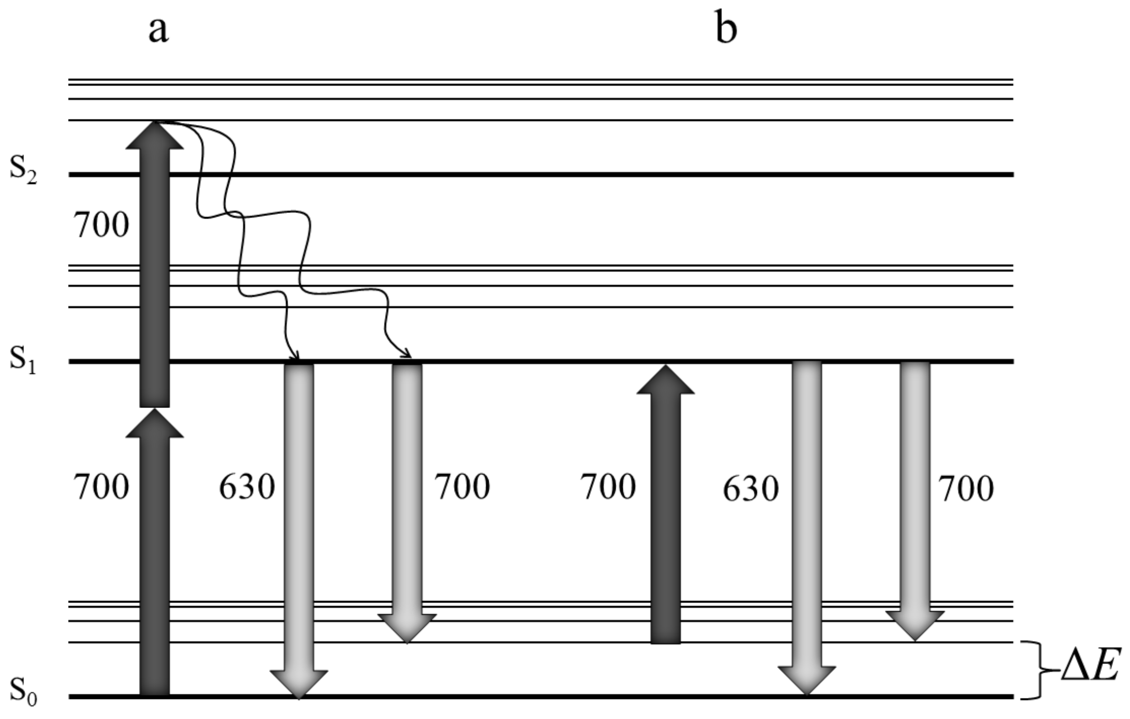

Figure S8. Schematic illustration of the two competing processes (a) two-photon excitation and (b) one-photon anti-Stokes fluorescence. Bold arrows represent absorption; grey arrows, emission; and curved arrows, radiation-less transitions. The population of the first vibronic level depends on the energy difference ΔE, described as the difference between the Q(0,0) and Q(0,1) emission.

The Bolzmann's distribution gives the thermal population, i.e. the proportion of molecules residing in the first vibronic level of S_0, given as:

$$p_{v=1} = \frac{N_{v=1}}{N_{v=0}} = e^{\frac{-\Delta E}{k_B T}}$$

where k_B is the Boltzmann's constant, and T is the temperature. Based on the wavelength on the Q-band emissions, 630 nm and 700 nm respectively, the thermal population of the first vibronic level $p_{v=1}$ could be approximated to 5×10^{-4}, i.e. corresponding to 0.05%, at room temperature.

To compare the probability of the different processes, the absorption cross-sections and the photon flux also has to be considered. The probability of the one-photon excitation (1PE) vs. the two-photon excitation (2PE) can then be described as:

$$P_{1PE} = \sigma_{1PE} \cdot I \cdot p_{v=1}$$
$$P_{2PE} = \sigma_{2PE} \cdot I^2$$

The probability P is given as [1/s], the 1PE cross section σ_{1PE} has the unit [cm^2], 2PE cross section σ_{2PE} has the unit [cm^4s], and the photon flux, I, is given as [$1/cm^2s$]. Here we approximate the σ_{1PE} to be 10^{-15} cm^2 (1), and assume σ_{2PE} to be 25 GM = 25 x 10^{-50} cm^4s according to literature for similar porphyrins (2).

The photon flux, I, is dependent on the output of the laser and the numerical aperture (NA) of the objective lens, according to the equation:

$$I = \frac{E_{pulse}}{E_{h\upsilon} \cdot A_{focus} \Delta t_{pulse}}$$

where

$$A_{focus} = \pi \cdot r_{focus}^{\ 2} = \pi \left(\frac{1.22\lambda}{2NA} \right)^2$$

given that the radius of the focal area is expressed according to the Rayleigh criteria.

The average laser energy was measured at the objective lens and found to be 3 mW using 710 nm wavelength and 1% laser power (as similar to experiments). The pulsewidth Δt_{pulse} given as full width at half maximum (FWHM) was determined to be 60 fs at the laser, and broadened to 80 fs by measuring at the objective lens through a skin sample. These parameters were incorporated in the calculations in Matlab, setting the repetition rate f_{laser} to 80 ± 1 MHz, according to specifications of the Mai Tai laser.

The probabilities for the 1PE and 2PE processes, i.e. P_{1PE} and P_{2PE}, were calculated for NA in the range 0.9 – 1.2. Given the conditions above, this gives that the populations of the first vibronic state $p_{v=1}$ should be larger than 10^{-4} for P_{1PE} and P_{2PE} to be equal. This means that in the case of PpIX, where a population of the first vibronic state is expected to be 5 x 10^{-4}, one-photon anti-Stokes fluorescence will have probability in the same range as two-photon excitation when the excitation light using fs-pulsed NIR is lowered towards 700 nm. Thus, these calculations are in agreement with the gradual transition from two-photon towards one-photon excitation that was observed experimentally.

1. Theodossiou T, MacRobert AJ. Comparison of the photodynamic effect of exogenous photoprotoporphyrin and protoporphyrin IX on PAM 212 murine keratinocytes. Photochemistry and Photobiology. 2002;76(5):530-7.
2. Drobizhev M, Karotki A, Kruk M, Rebane A. Resonance enhancement of two-photon absorption in porphyrins. Chemical Physics Letters. 2002;355(1-2):175-82.
